# Degenerative Cervical Spondylosis: Natural History, Pathogenesis, and Current Management Strategies

**DOI:** 10.1155/2012/916987

**Published:** 2012-08-27

**Authors:** Joseph S. Butler, F. Cumhur Öner, Ashley R. Poynton, John M. O'Byrne

**Affiliations:** ^1^Department of Trauma and Orthopaedic Surgery, Royal College of Surgeons in Ireland, Cappagh National Orthopaedic Hospital, Finglas, Dublin 11, Ireland; ^2^Department of Orthopaedic Surgery, University Medical Centre Utrecht, Heidelberglaan 100, 3584 CX Utrecht, The Netherlands; ^3^Department of Orthopaedic & Spinal Surgery, Mater Private Hospital, Eccles Street, Dublin 7, Ireland

Degenerative cervical spondylosis is a common, mostly asymptomatic condition, occurring as a result of age-related degenerative changes in the cervical spine. Symptoms caused by cervical spondylosis can be categorized broadly into three clinical syndromes: axial neck pain, cervical radiculopathy, and cervical myelopathy; with patients commonly having a combination of these syndromes. This special issue contains eleven papers summarizing our present knowledge and understanding of the natural history, pathogenesis, and current management strategies for degenerative cervical spondylosis.

In the paper entitled “*The natural history and clinical syndromes of degenerative cervical spondylosis*,” J. C. Kelly et al. outline the three clinical syndromes of axial neck pain, cervical radiculopathy, and cervical myelopathy. Radiographic evidence of spondylotic changes is frequently found in many asymptomatic adults. The majority of symptomatic patients present between the ages of 40 and 60, with men more commonly affected than women at a ratio of 3 : 2. Disc degeneration and bulging, osteophyte and spur formation, ligamentous hypertrophy, vertebral subluxation, decreased disc height, and facet joint arthropathy may all contribute to narrowing of the spinal canal and intervertebral foramina. Radiculopathy is a result of intervertebral foramina narrowing. Narrowing of the spinal canal results in spinal cord compression, ultimately resulting in cervical myelopathy. The course of disease development and the ultimate prognosis for patients with cervical spondylosis is highly variable and extremely difficult to predict.

In the paper entitled “*The natural history and clinical presentation of cervical spondylotic myelopathy*,” C. K. Yarbrough et al. describe cervical spondylotic myelopathy as an impaired function of the spinal cord caused by degenerative changes of the cervical spine resulting in spinal cord compression. While many patients with mild signs of cervical spondylotic myelopathy will stabilize or improve over time with conservative management, the clinical course of a specific individual patient cannot be predicted. Asymptomatic patients with cervical stenosis and abnormalities on electrophysiologic studies may be at higher risk for developing myelopathy.

L. A. Ferrara identifies aging as the major risk factor contributing to the onset of cervical spondylosis in the paper “*The biomechanics of cervical spondylosis*.” Several acute and chronic symptoms can occur that start with neck pain and may progress into cervical radiculopathy. Eventually, the degenerative cascade causes desiccation of the intervertebral disc resulting in height loss of the cervical spine. This causes ventral angulation and eventual loss of lordosis, with compression of the neural and vascular structures. The altered posture of the cervical spine progresses into kyphosis if the load balance and lordosis is not restored.

C. Green et al. in their paper entitled “*Imaging modalities for cervical spondylotic stenosis and myelopathy*,” highlight the central role of diagnostic imaging in clinical diagnosis and preoperative planning. Magnetic resonance imaging (MRI) provides the greatest range of information compared with other radiological studies available to evaluate the spine. It provides an accurate morphological assessment of both osseous and soft tissue structures including intervertebral discs, spinal ligaments, and the neural elements. Dynamic weight-bearing MRI has recently been championed as the preferred technique for pathology-specific diagnosis. Computed tomography in isolation lacks the soft tissue detail achieved with MRI scanning, however, is still a useful modality when there is a contraindication to MRI and where metal artefact is obstructing the anatomy. CT myelography is an invasive procedure, associated with a number of risks, and is only used for patients with contraindications, equivocal findings, or failed MR imaging because of metal artefact.

In the paper entitled “*Nonoperative modalities to treat symptomatic cervical spondylosis*,” K. M. Hirpara et al. state that cervical spondylosis is a common and disabling condition. It is generally felt that the initial management should be nonoperative, and these modalities include physiotherapy, analgesia, and selective nerve root injections. Surgery should be reserved for moderate to severe myelopathy patients who have failed a period of conservative treatment and patients whose symptoms are not adequately controlled by nonoperative means. The authors conclude that effective, nonoperative treatment is labour intensive, requiring regular review and careful selection of medications and physical therapy on a case-by-case basis.

Yalamanchili et al. describe in their paper “*Cervical spondylotic myelopathy: factors in choosing the surgical approach*,” the variety of surgical options that exist, including anterior and posterior approaches with and without fusion. Systematic review does not clearly show one technique to be clinically superior to another. Therefore decision-making depends on individual patient factors and associated approach-related complications. Factors to consider include location of cord compression, number of levels involved, sagittal alignment, instability, associated axial neck pain, and risk factors for pseudoarthrosis.

The paper entitled “*Operative techniques for cervical radiculopathy and myelopathy*,” R. G. Kavanagh et al. state that surgical decompression can be achieved through a multitude of procedures using either an anterior or posterior approach. The main procedures that are performed through an anterior approach are anterior cervical discectomy and corpectomy, and those carried out through a posterior approach are laminoplasty, laminectomy, and posterior cervical discectomy. The type of procedure carried out is dependent on a number of different variables including extent and location of pathology, previous surgery, congenital canal stenosis, and the presence of preoperative axial neck pain. Satisfactory surgical outcome will result in long-term amelioration of cervical radiculopathic and myelopathic symptoms with few postoperative complications.

The paper entitled “*Operative techniques for cervical radiculopathy and myelopathy*,” C. Moran and C. Bolger. suggest that surgical outcome is dependent on selecting the appropriate treatment for the appropriate patient and pathology. Once the decision is made to manage the patient operatively, the principal decision is whether to choose the ventral or the dorsal approach. In cervical spondylosis, several variables including the location of pathology (ventral, dorsal, circumferential), extent of pathology (limited to interspace, extensive behind vertebral body), the number of levels affected, the presence of instability or the presence of kyphotic deformity require consideration. In general, any procedure chosen should decompress the affected spinal cord or nerve roots, maintain or restore stability, and correct or prevent kyphotic deformity.

B. A. Braly et al. describe the technique of laminoplasty as a motion-sparing posterior decompressive method in “*Operative treatment of cervical myelopathy: cervical laminoplasty*.” The authors describe the techniques of open-door or “hinged” laminoplasty. Laminoplasty or decompression, with retention of the posterior elements, offers the surgeon multiple advantages as a treatment option. The idea of a motion-sparing technique is the largest benefit when comparing laminoplasty to laminectomy and posterior fusion. Although complications may still occur and special care must be paid to patient selection, laminoplasty is a viable option to consider when treating patients with cervical myelopathy.

In the paper entitled “*Laminoplasty techniques for the treatment of multilevel cervical stenosis*,” L. K. Mitsunaga et al. state that laminoplasty is becoming an increasingly popular technique for the treatment of multilevel cervical stenosis due to cervical spondylotic myelopathy, OPLL, and other causes. It minimizes the risk of certain complications associated with other surgical options, such as graft and fusion-related complications, postoperative kyphosis and instability, and the morbidity of an anterior approach. It does, however, have its own set of potential complications, including laminar closure, axial neck pain, nerve root palsies, and loss of cervical motion and alignment. Laminoplasty techniques are continuously being refined to address such potential shortcomings. Outcomes from laminoplasty are at least as good as anterior decompression and fusion or laminectomy and fusion. In the appropriate patient and with proper surgical technique, laminoplasty is a good option for patients with multilevel cervical stenosis and myeloradiculopathy.

In the paper entitled “*Operative outcomes for cervical myelopathy and radiculopathy*,” J. G. Galbraith et al. state that when considering surgical outcomes for cervical myelopathy, it is important to remember that regardless of surgical technique employed, results of operative treatment generally are better in patients who undergo early decompression. Patients with less than a one-year duration of symptoms show significantly greater motor recovery following operation than did those with a longer duration of symptoms. Conversely, the symptoms for most patients with degenerative cervical radiculopathy will be self-limited and will resolve spontaneously over a variable length of time without specific treatment. Surgical intervention, however, can lead to rapid relief of symptoms of cervical radiculopathy compared to conservative measures alone. At present, there is insufficient evidence to indicate whether anterior or posterior surgery yields superior short- and long-term results for either cervical myelopathy or radiculopathy.

We expect that this special issue will help the surgeons to make sound decisions on the treatment of these increasingly common problems in our rapidly aging population.

*Joseph S. Butler* *F. Cumhur Öner* *Ashley R. Poynton* *John M. O'Byrne*

